# To comply or not comply? A latent profile analysis of behaviours and attitudes during the COVID-19 pandemic

**DOI:** 10.1371/journal.pone.0255268

**Published:** 2021-07-29

**Authors:** Sabina Kleitman, Dayna J. Fullerton, Lisa M. Zhang, Matthew D. Blanchard, Jihyun Lee, Lazar Stankov, Valerie Thompson

**Affiliations:** 1 School of Psychology, University of Sydney, Sydney, Australia; 2 School of Education, University of New South Wales, Sydney, Australia; 3 Department of Psychology, University of Saskatchewan, Saskatoon, Canada; University of Haifa, ISRAEL

## Abstract

How and why do people comply with protective behaviours during COVID-19? The emerging literature employs a variable-centered approach, typically using a narrow selection of constructs within a study. This study is the first to adopt a person-centred approach to identify complex patterns of compliance, and holistically examine underlying psychological differences, integrating multiple psychology paradigms and epidemiology. 1575 participants from Australia, US, UK, and Canada indicated their behaviours, attitudes, personality, cognitive/decision-making ability, resilience, adaptability, coping, political and cultural factors, and information consumption during the pandemic’s first wave. Using Latent Profile Analysis, two broad groups were identified. The compliant group (90%) reported greater worries, and perceived protective measures as effective, whilst the non-compliant group (about 10%) perceived them as problematic. The non-compliant group were lower on agreeableness and cultural tightness-looseness, but more extraverted, and reactant. They utilised more maladaptive coping strategies, checked/trusted the news less, and used official sources less. Females showed greater compliance than males. By promoting greater appreciation of the complexity of behaviour during COVID-19, this research provides a critical platform to inform future studies, public health policy, and targeted behaviour change interventions during pandemics. The results also challenge age-related stereotypes and assumptions.

## Introduction

On March 11^th^ 2020, the World Health Organization (WHO) characterised the novel coronavirus outbreak a global pandemic [1]. Subsequently, governments and health authorities called on citizens to adopt protective behaviours such as social distancing, hygiene practices, and self-isolation to reduce the spread of coronavirus disease 2019 (COVID-19). Implementation of restrictions such as border closures and lockdowns have been driven by policy, system, and environmental changes. However, the success of these measures relies on individual behaviour change. Understanding the factors associated with positive behaviour change is critical as the virus spreads.

Research on previous health threats has identified demographic and attitudinal factors influencing adherence with protective behaviours, such as age, gender, education, worry and fear, greater perceived risk, and belief in effectiveness of behaviors [e.g., 2–4; see 5 for review]. Attesting to their validity, emerging research on COVID-19 suggests these factors are indeed associated with adherence to rules and recommendations [e.g., 6–10].

These emerging studies take a variable-centered approach, assuming the sample is homogeneous with respect to how variables relate to each other. The current research adopts an alternative perspective, focusing on a person-centred approach, which acknowledges heterogeneity within the sample, offering the means to identify clusters/groups of individuals who share similarities. These groups can then be examined further to characterise their psychological and environmental profiles [11]. In relation to the COVID-19 pandemic, this novel approach is much needed, though, to our knowledge has not yet been explored.

This research was conceived and executed during the first wave of COVID-19, which was characterised by extreme uncertainty about the virus and rapid, radical changes to people’s lives. Our main objective was to identify subgroups within the general population with similar patterns of COVID-19-related behaviours and attitudes, using Latent Profile Analysis (LPA). Additionally, this study examined differences between identified subgroups (i.e., those who comply and those who do not) on a comprehensive selection of factors grounded in several disciplines of psychological sciences—epidemiology, differential (personality, cognitive ability), positive (resilience, adaptability, coping), and political and cultural (government opinions, ideologies, cultural tightness-looseness). This allowed us to examine the prevalence of distinct groups and their characteristics to inform targeted public health policies and behaviour change interventions for the current and future pandemics. To our knowledge, this is the first study to capture such a large selection of theory-driven constructs, one that acknowledges both the complexity of the effect of the pandemic on behaviour, as well as the interaction with individual characteristics. Our goal was to draw from a range of theoretical positions to achieve a more holistic account of compliance behaviours. Below, we outline the rationale for examining the relationships between a broad set of variables, ranging from personality dimensions and cognitive abilities to information consumption and political values, and compliance behaviours during COVID-19.

### Behaviours and attitudes underlying compliance during COVID-19

We begin with an examination of behaviours and attitudes that may underlie compliance with public health advice during the current pandemic. Early research into the H1N1 pandemic [5] suggested that there are three types of protective behaviours: preventive (e.g., hygiene practices), avoidant (e.g., social distancing, isolation), and management of illness (e.g., seeking medical help if unwell). In the present study, we asked participants about their adoption of behaviours falling under these three categories which generally align with COVID-19 rules and recommendations. Moreover, changes to these behaviours may be driven by emotional perceptions such as heightened worry, fear, and anxiety about the virus. Research from past outbreaks (SARS, Avian influenza) found that higher worry and state anxiety were associated with adopting protective behaviours such as hand-washing [2–4, 12]. This is consistent with emerging COVID-19 research [6, 8]; whereby worry and fear of COVID-19 were primary predictors of positive behaviour change.

These findings are consistent with health behaviour theories such as Protection Motivation Theory [13]. This theory proposes that four components facilitate behaviour change: perceived severity of the threat, perceived vulnerability to infection, perceived efficacy of protective behaviours, and perceived self-efficacy in executing behaviours [13]. Similarly, the Health Belief Model suggests greater perceived susceptibility to and severity of the threat, and perceived benefits and barriers of protective actions predict health behaviour adoption [14]. Hence, we employed measures of worry about COVID-19, as well as attitudes and beliefs about protective measures to examine the role of attitudes in compliance with COVID-19 protective behaviours.

Additionally, the pandemic has resulted in behaviours that can loosely be described as pro- and anti-social. For instance, in some, the pandemic has driven individualistic and competitive behaviours, such as panic buying, and hostile disagreements; whilst in others, the shared challenges have fostered a sense of collectiveness, facilitating altruistic behaviours such as donations and offering support to others. To examine the relationships between such behaviours and compliance, we asked participants to report their engagement with a range of pro- and anti-social behaviours over the past week.

### Factors associated with behaviours and attitudes towards compliance

#### Gender

A meta-analysis of the relationship between gender and protective behaviours during respiratory epidemics and pandemics found that females were about 50% more likely than men to adopt behaviours such as hand-washing and avoidance of public transport [15]. On the other hand, males were more likely to support pharmaceutical measures such as vaccination [15]. This suggests there are inherent differences between how females and males respond to virus outbreaks, which were examined in this study.

#### Personality and cognitive abilities

Differential psychology also offers insight into systematic tendencies towards compliant behaviours and attitudes. Individual differences in Big-5 traits and cognitive abilities may account for some of the diversity in compliance; but to date, the data is sparse and results are mixed. This research was conducted during the first wave of the pandemic before the emerging findings. The inclusion of personality and cognitive ability measures allows us to clarify and extend these findings.

Conscientiousness is associated with following norms and rules, self-discipline, and ability to delay gratification [e.g., 16]; and has emerged as a consistent predictor of positive health behaviours [17]. Those high in agreeableness tend to be cooperative and concerned for others [e.g., 16], thus might be more inclined to adhere to rules and recommendations. Emerging research has found support for these notions in relation to COVID-19 recommendations, including social distancing and hygiene [e.g., 18, 19]. Extraverted people are generally more sociable [e.g., 16], meaning adherence with social distancing might require a greater lifestyle change than those who are introverted. However, the emerging findings related to COVID-19 have been mixed. Aschwanden et al. [18] found extraversion to be associated with taking more precautions such as hand-washing, sanitising, and physical distancing; whilst Blagov [19] found no relationship between extraversion and social distancing or hygiene behaviours after controlling for other traits. Similarly, the relationship between neuroticism and compliance is unclear as those high on this trait tend to engage in certain health-risk behaviours but are also predisposed to elevated anxiety and worry which may facilitate adoption of health-promoting behaviours. The emerging COVID-19 findings are indeed inconsistent. For instance, Aschwanden et al. [18] found that neuroticism was associated with fewer precautionary behaviours, whilst Blagov [19] found that neuroticism played a small positive role in current social distancing behaviour, as well as intention to adopt hygiene behaviours in future; though no relationship with intention to engage in social distancing in future. Lastly, people high on openness/intellect are characterised by being adept at dealing with change and uncertainty [e.g., 16], whilst also being risk-takers; as such, it is unclear how this trait relates to compliance with protective measures. Aschwanden et al. [18] found openness was associated with taking more precautions, washing hands, avoiding touching, and physical distancing; whilst Blagov [19] found no association between openness and COVID-19 protective behaviours. Whilst conscientiousness and agreeableness have emerged as consistent predictors of positive health behaviours, results concerning extraversion, neuroticism, and openness/intellect require further clarification. Hence, we captured the Big 5 traits, allowing us to clarify and extend findings in relation to personality.

Complying with recommendations requires individuals to prioritise future rewards (e.g., flattened curve) over immediate rewards of non-compliance (e.g., visiting friends, partaking in hobbies). Frederick [20] found support for the notion that those with a disposition to analytical thinking are less likely to discount the value of future rewards. That is, people scoring higher on a cognitive reflection test were more likely to choose a larger reward in the short-term future over an immediate, smaller reward than low-scoring people. Scores on this test are correlated with measures of cognitive ability [20, 21], leaving open the possibility that cognitive and decision-making abilities play a role in willingness to make immediate sacrifices for longer term gains. Xie et al. [10] found that working memory capacity contributes to social distancing compliance, which they suggest may be explained by the relationship between working memory and ability to accurately evaluate the benefits of social distancing guidelines. This finding is in line with the idea that the ability to prioritise benefits over costs plays a role in compliance with protective behaviours; however, there is little data investigating this during COVID-19. To this end, we included the Cognitive Reflection Test as well as two other cognitive ability tasks, the Esoteric Analogies Test and Syllogistic Reasoning Task, to investigate how performance on these tasks relates to compliance during COVID-19.

#### Resilience, adaptability, coping

Emerging research has documented the detrimental consequences of COVID-19 on mental health, including increased stress, anxiety, depression, loneliness, and uncertainty about the future [see 22, 23]. In particular, reduced social and physical contact resulting from restrictions and lockdown measures may have major psychological impacts, thwarting willingness to comply with protective measures, particularly avoidant ones. Coming from the positive psychology literature, the constructs of resilience, adaptability, and coping explore how people respond and adapt in the face of uncertainty and adversity, potentially protecting individuals against these negative consequences [see 24–26 for reviews]. Emerging research provides some confirmation of these predictions, with Sibley et al. [27] finding that those high in resilience in a New Zealand sample showed minimal short-term detrimental effects of lockdown. Whilst this preliminarily demonstrates the important role resilience plays in mental well-being during COVID-19, it is unclear whether higher self-perception of resilience and adaptability would foster compliant behaviour. There is also a lack of research examining the relationship between coping strategies and compliance behaviour. The current study addresses this gap and extends upon existing literature on the mental health consequences of COVID-19 protective measures by including measures of resilience, adaptability, and strategies for coping with the COVID-19 pandemic.

#### Political and cultural variables

Cultural and political psychology integrate individual- and group-level differences to understand social behaviour, thus should provide valuable insights into how people adjust their social behaviour to comply with protective measures. For instance, social conservatism describes a political ideology that favours tradition and maintaining the status quo [28]. Relatedly, right-wing authoritarianism encompasses support for tradition, control, and willingness to submit to authority [29]. These social attitudes may shape perceptions of protective measures, as well as government and authority figures, and the messages they deliver; though their role in compliance has yet to be examined. Conversely, reactance theory suggests that when individuals feel restricted, they feel the urge to resist control and regain their freedoms [30]. In particular, reactance may be stronger in ‘looser’ cultures, which prioritise freedom and have greater tolerance for deviance [31]. On the other hand, ‘tight’ cultures are more accustomed to strict social norms and rule enforcement. Van Bavel et al. [32] suggest that different strategies for containing COVID-19 might be needed depending on the cultural context; though the relationship between reactance, tightness-looseness and compliance behaviour is yet to be investigated. This study addresses this gap by capturing ideologies, reactance tendencies, and tightness-looseness perceptions, and examining their relationships with COVID-19 protective behaviours.

Lastly, for the majority who are not part of the at-risk, vulnerable population, compliance requires a degree of prioritising the protection of others over one’s own self-interest. Thus, we also captured amoral social attitudes, including disregard for others and rules. To our knowledge no study published to date has reported findings on amoral social attitudes. It is important, however, to clarify whether non-compliance might be driven by self-interest, more than any other factor.

#### Information consumption

We also examined how people obtained information about the pandemic. Information plays a central role in forming our knowledge, attitudes, and beliefs. An analysis of broadcast news segments addressing COVID-19 in January and February revealed the most mentioned topics were death-related, whilst only a minority provided information about preventing the spread of the virus [33]. These messages may arouse negative emotions, which may motivate protective behaviour. On the other hand, however, they may miss the opportunity to educate the public on adaptive ways to respond. Hence, it is unclear what role frequency of checking the news plays in motivating behaviour change. This study sought to clarify this relationship. We also examined the outlets that people used (e.g., official vs. informal), and whether or not people sought to verify the accuracy of information (e.g., to prevent the spread of misinformation and fake news).

Further, the way information is received and acted on depends on level of trust in the government and authority figures from which the messages come. The emerging research suggests that those with greater trust in government and authorities are more likely to report compliance with hygiene and avoidance behaviors [9]. To further examine how government perceptions relate to compliance, this study captured perceptions of government truthfulness, satisfaction with the government’s response to the COVID-19 outbreak, and appropriateness of the government’s reaction.

In sum, this research examined several different aspects of information consumption: frequency, sources, tendencies to verify the accuracy of information, and attitudes towards government, aiming to provide valuable insights into how media use and consumption influence compliance behaviour. This understanding can help in building recommendations about how to communicate pandemic related messages effectively.

#### Future behaviours

We also captured people’s behavioural intentions by asking them to indicate the reasons they expect to leave home for in the following week [34]. Whilst some (e.g., going to work, going to the pharmacy) are legitimate reasons for leaving home, others defy stay-at-home orders (e.g., meeting friends or relatives, getting bored). This was not intended as a measure of compliance, but rather to provide a greater understanding of why people are non-compliant with avoidant protective measures, and differences in reasons between compliant and non-compliant groups. This is important for informing effective behaviour change strategies which might address and accommodate for the reported reasons for not staying home or social distancing.

### International sampling

We drew a large sample from four English-speaking countries: United States (US), United Kingdom (UK), Australia, and Canada. Although these countries share similar language, and social and cultural norms, the national governments of these four countries took varied approaches in responding to COVID-19. Hence, country-level differences were examined.

### The present study

The literature reviewed above has adopted a variable-centered approach to understanding compliance, where the focus is on how variables relate to each other. By contrast, we adopted a person-centred approach by using Latent Profile Analysis (LPA) to identify clusters/groups of people within the general population who share similar patterns of COVID-19 behaviours and attitudes and determine their situational and psychological profiles. This approach acknowledges heterogeneity within the sample, providing important insights into how subgroups within the sample differ from one another. Although LPA is largely a data-driven process, our variables were theoretically drawn from a wide spectrum of psychology paradigms. The first aim was to identify subgroups of individuals who clustered together on COVID-19-related behaviours and attitudes, along with key demographics (age, education, physical health, and pre-existing health conditions). The second aim was to examine differences between the subgroups (i.e., compliant and non-compliant), on personality, cognitive and decision-making abilities, coping, information consumption, and cultural and political factors. The final aim was to examine country and gender differences within subgroups. To the best of our knowledge, this is the first study to provide a comprehensive psychological profile of compliant and non-compliant groups of individuals. The pandemic is multi-faceted and complex, and our approach reflects this complexity by integrating the literature in a holistic manner, which allows us to combine multiple pieces of the puzzle, rather than focusing on a single, possibly critical, but narrow, piece.

## Method

### Participants and procedure

Participants were recruited via snowball sampling (n = 290), a University participant recruitment system (n = 415), and Prolific (n = 870). The final sample comprised 1575 participants, including 609 (38.7%) from Australia (76.19% female, Mean age = 25.95, SD = 12.64); 366 (23.2%) from UK (66.39% female, Mean age = 35.21, SD = 14.51); 303 (19.2%) from Canada (50.50% female, Mean age = 30.88, SD = 10.41); and 297 (18.8%) from US (54.88% female, Mean age = 32.77, SD = 12.40). An additional 103 participants from non-English speaking countries participated via social media recruitment, including Afghanistan (n = 1), Argentina (n = 1), Belgium (n = 1), China and Hong Kong (n = 16), Czech Republic (n = 1), France (n = 1), Germany (n = 23), Hungary (n = 1), Indonesia (n = 2), Republic of Ireland (n = 1), Israel (n = 8), Japan (n = 1), Kyrgyzstan (n = 1), Malaysia (n = 1), Netherlands (n = 4), Norway (n = 2), Philippines (n = 5), Republic of Korea (n = 5), Serbia (n = 2), Singapore (n = 7), Slovenia (n = 1), South Korea (n = 4), Sweden (n = 1), Thailand (n = 2), Macedonia (n = 1), and Taiwan (n = 10). Limited sample size within each of these countries precludes the ability to provide comparisons, thus they are not included in this paper. An additional 46 people with a large number of missing values (above 40%, with most above 80%) were deleted prior to analyses. For the 1575 included participants, random missing data was imputed using the EM procedure prior to LPA. Non-random missing data was not imputed and relevant degrees of freedom are reported in relevant tables. Those recruited via snowballing participated voluntarily. University students participated in return for course credit. Participants recruited from Prolific were compensated with approximately £2.50. The study was accessed online via Qualtrics. Data was collected between 1^st^ April and 20^th^ May 2020, during the first wave of the pandemic when recommendations and restrictions in the four countries sampled were generally to socially distance, adopt hygiene measures including hand-washing and sanitising, stay at home except for essential reasons (e.g., health care, essential work, physical exercise, shopping for essentials), and self-isolate and get tested if experiencing any symptoms. Participants completed an online survey which took approximately 30 to 40 minutes. They first answered questions about information consumption, followed by a demographics questionnaire consisting of age, gender, birth country, country of residence, educational attainment, political orientation (from 1 = extremely conservative to 7 = extremely liberal), physical health level (from 0 = poor to 4 = excellent), and number of existing health conditions. They then completed the Cognitive Reflection Test, followed by measures of resilience, COVID-19 behaviours, syllogistic reasoning, adaptability, COVID-19 worry, intelligence, COVID-19 attitudes and beliefs, coping, Big 5 personality, cultural tightness-looseness, right-wing authoritarianism, social conservatism, amorality, and reactance. Ethics approval was granted by the University of Sydney Human Research Ethics Committee (protocol number 2020/184) and the University of Saskatchewan Behavioural Research Ethics Board (ID 1944).

### Materials

In addition to using validated measures to capture constructs outlined in the introduction, new scales were developed to capture COVID-19 behaviours and attitudes. Table 1 summarises all measures used. This study was part of a larger COVID-19 research project. Data collected for the Australian sample included additional measures which are outside the scope of this paper.

**Table 1:** Measures employed in this study.

Measure (Authors)	Number of items and response scale	Dimensions and example items	Internal consistency (previous studies)
**LPA Profile Indicators: COVID-19 Measures**			
Self-Report Compliance (developed for this study, see Appendix A in S1 Appendix)	4 items (1) *strongly disagree* to (5) *strongly agree*	“I follow my government’s restrictions to protect myself from COVID-19”	-
Protective Behaviours (adapted from [34], see Appendix A in S1 Appendix)	12 items (0) *does not apply at all* to (100) *applies very much*	Preventive: “I washed my hands more often” Avoidant: “I stayed home” Management of illness: “If I had exhibited symptoms of sickness, I would have immediately called a doctor”	-
Prosocial and Antisocial Behaviours (developed for this study, see Appendix A in S1 Appendix)	8 items (0) *does not apply at all* to (100) *applies very much*	Prosocial: “Provided more emotional support to strangers” Antisocial: “Bought more products (e.g., groceries) from the supermarket than you normally would”	-
COVID-19 Worry [34]. This measure assessed concerns related to COVID-19.	5 items (1) *does not apply at all* to (5) *strongly applies*	“I am nervous when I think about current circumstances”	-
COVID-19 Beliefs (developed for this study and adapted from [34], see Appendix A in S1 Appendix).	10 items (1) *strongly disagree* to (5) *strongly agree*	Response Efficacy: “Social distancing is effective in slowing the spread of COVID-19” Perceived Benefits: “People should cancel their participation at social gatherings right now” Perceived Barriers: “Social distancing will likely destroy our economy”	-
**LPA Profile Indicators: Demographic characteristics**			
Age	1 item	What is your age?	-
Gender	1 item	Which gender do you identify with?	-
Physical Health	1 item (1) *poor* to (4) *excellent*	How physically healthy are you?	-
Pre-Existing Health Conditions	1 item	Consider the following list of health conditions: immunosuppressed conditions, cardiovascular diseases, diabetes, hepatitis B, chronic obstructive pulmonary disease, chronic kidney diseases, and cancer. How many of these conditions do you have?	-
**Personality Measure**			
Mini International Personality Item Pool [35]	20 items (1) *very inaccurate* to (5) *very accurate*	Extraversion: “I am the life of the party” Agreeableness: “I sympathize with others’ feelings” Conscientiousness: “I get chores done right away” Neuroticism: “I have frequent mood swings” Intellect/Openness: “I have a vivid imagination”	.65 to .82 [35]
**Cognitive and Decision-Making Abilities**			
Esoteric Analogies Test [36]	20 items	LIGHT is to DARK as HAPPY is to: GLAD, SAD*, GAY, EAGER	.64 and .76 [37, 38]
Cognitive Reflection Test [39]	7 items	A bat and a ball cost $1.10 in total. If the bat costs $1.00 more than the ball, how much does the ball cost? [from 20]. Jerry received both the 15^th^ highest and the 15^th^ lowest mark in the class. How many students are in the class?	.72 [39]
Syllogistic Reasoning Task [40]	8 items	Decide whether the conclusion follows logically from the premises. Premise 1: All flowers have petals. Premise 2: Roses have petals. Conclusion: Roses are flowers.	.86 [41]
**Resilience, Adaptability, Coping Measures**			
Connor-Davidson Resilience Scale Short Version [42]	10 items (0) *not true at all* to (4) *nearly always true*	“I can deal with whatever comes”	.85 [42]
Individual Adaptability Scale [26].	15 items (1) *strongly disagree* to (5) *strongly agree*	Two subscales were included in this study: Handling Crises: “I am able to maintain focus during emergencies” Tolerance for Uncertainty: “I perform well in uncertain situations”	.74 to .81 [43]
Brief COPE Inventory [44]	28 items (1) *I haven’t been doing this at all* to (4) *I’ve been doing this a lot*	14 subscales “I’ve been turning to work or other things to take my mind off things”	.50 to .90 [44]
**Political and Cultural Measures**			
Government Truthfulness [34]	1 item (1) *very untruthful* to (5) *very truthful*	“How factually truthful do you think your country’s government has been about the COVID-19 outbreak?”	-
Government Satisfaction [34]	1 item (1) *very dissatisfied* to (5) *very satisfied*	“How satisfied are you with your country’s government response to the COVID-19 outbreak?”	-
Government Reaction [34]	1 item (1) *not at all sufficient* to (5) *much too extreme*	“Do you think the reaction of your country’s government to the COVID-19 outbreak is appropriate, too extreme, or insufficient?”	-
Conservatism Scale [45]	3 items were selected from the 12-item scale. This is a measure of social conservatism [28] (1) *fully disagree* to (5) *fully agree*	“We have to respect our history and tradition”	-
Right-Wing Authoritarianism Scale [46]	3 items (1) *fully disagree* to (5) *fully agree*	“We should take strong action against misfits and slackers in society”	.74 to .86 [46]
Cultural Tightness-Looseness Index [31]	6 items (1) *strongly disagree* to (6) *strongly agree*	“There are many social norms that people are supposed to abide by in this country”	.85 [31]
Hong Psychological Reactance Scale [47]	14 items (1) *strongly disagree* to (6) *strongly agree*	“Regulations trigger a sense of resistance in me”	.75 to .80 [48]
Amoral Social Attitudes [49]	6 items (1) *fully disagree* to (5) *fully agree*.	“I hate obligations and responsibilities of any kind”	-
**Other COVID-19 Measures**			
Information Consumption (developed for this study, see Appendix A in S1 Appendix)	7 items (1) *never* to (5) *all the time*	Which sources do you get information about COVID-19? Formal Sources: “Official Government websites” Casual Sources: “Social media”	-
Future Behaviours [34]	11 items (1) *multiple times a day* to (5) *not at all*	What are the reasons for you to leave home in the next week? e.g., going to work, walking a pet	-

### Statistical analysis

**Fig 1:**
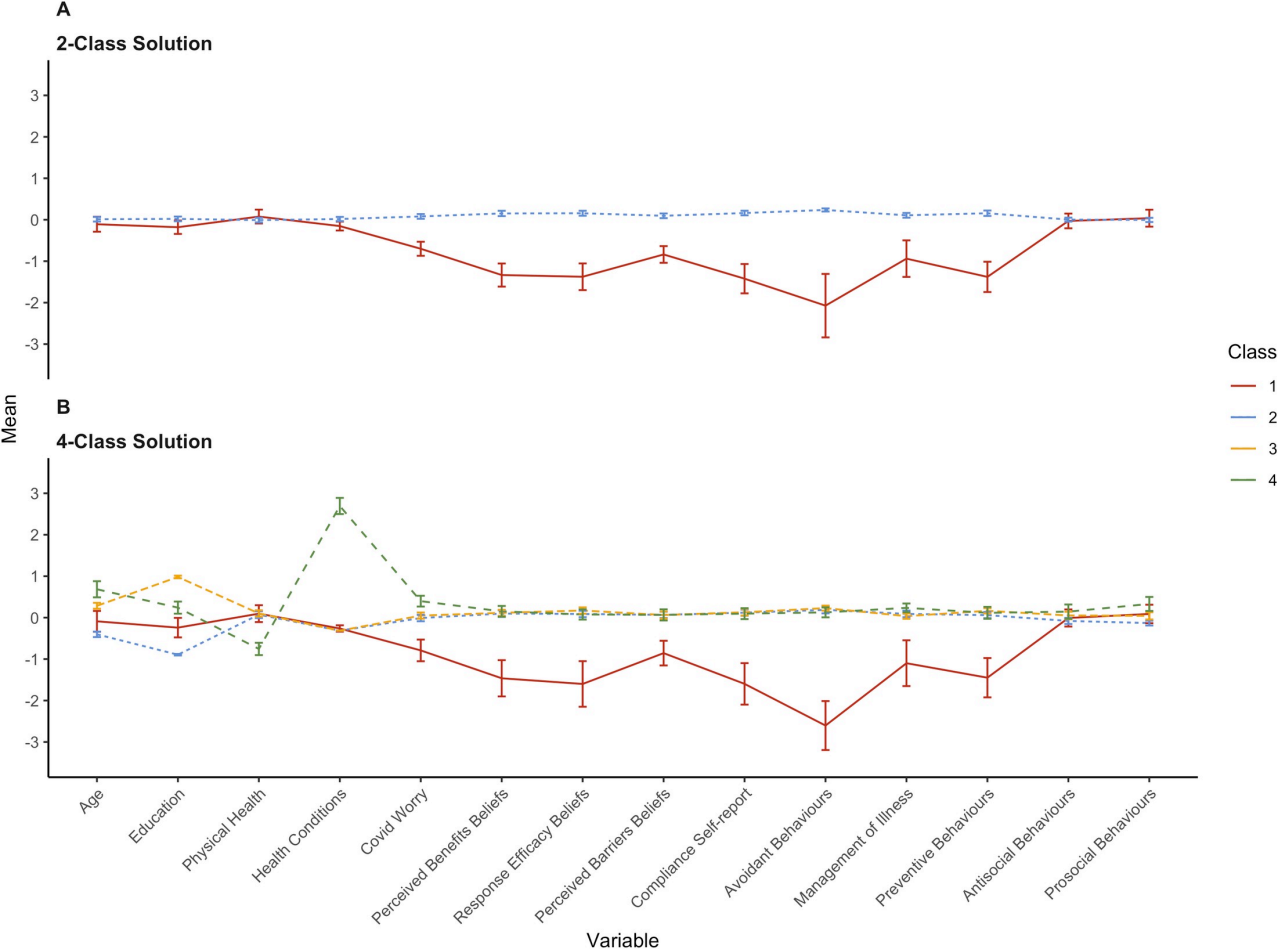
Latent profile groups for 2- (A) and 4-class (B) solutions. Error bars represent the standard error of the mean.

14 variables including COVID-19 beliefs and behaviours, and demographic characteristics (see Table 1 and Fig 1) were analysed using LPA to reveal potential groupings of individuals sharing similar patterns of responses. To enable direct comparisons, variables were standardised. A series of independent samples *t*-tests were conducted to examine differences between the resulting profiles on measures capturing psychological factors, information consumption, political and cultural factors, and future behaviours. To estimate power required, we employed a typical heuristic for multivariate data analysis; a minimum of 10, but preferably 20 cases per variable in the model [50, see also 51 for a review on LPA]. Thus, the sample size estimate was a minimum of 280 participants in each country. Given time constraints, some measures (cognitive ability and decision-making) were only collected for some individuals. Degrees of freedom are listed in tables in the Results section. R and Mplus statistical software packages were used for all analyses.

## Results

### Latent Profile Analysis (LPA)

#### Selection of a solution

Both Latent Profile (LPA) and cluster analyses were conducted. LPA and cluster analysis both focus on classifying the observations under study into groups given their homogenous characteristics across a set of estimated values of the predictor variables. While the objectives are the same, the two approaches differ in several ways. What LPA can do whilst clustering methods cannot is produce a set of ‘profiles’ in which each group will have its own ‘profile’ consisting of the estimated values on the predictor variables. The ‘final’ modelling outcome of clustering methods is a hierarchical grouping of the units of analysis, usually presented as a dendrogram. An additional benefit of LPA over clustering is the broad range of fit indices that can be used to assess model fit and determine the optimal number of profiles. Thus, we adopted LPA. Still, given that LPA and cluster analysis may produce different results [52], we also performed a hierarchical cluster analysis to check that the results were robust. The cluster analysis results were consistent with those of the LPA in two important ways: identifying the same two clusters (compliant and non-compliant) and identifying people within each cluster.

LPA was performed for 2–6 class solutions, with 1 class as the default (see fit statistics in Table 2). With the exception of entropy, a clear trend was observed for the model fit statistics (AIC, BIC, adjusted BIC, and log-likelihood): solutions with a higher number of classes demonstrated better model fit. This was further evidenced by the log-likelihood test indicating that, as the number of classes increased, the improvement in model fit was statistically significant for each successive solution. This was expected as a higher number of fitting parameters, in principle, should lead to a better fit to the data than a smaller number of parameters.

**Table 2:** Latent profile analysis based on COVID-19 behaviours and attitudes.

Classes in the model	AIC	Adjusted BIC		BIC		Entropy		LogL	df		*p*-value
**Class 2**	**60585**	**60679**		**60816**		**0.952**		**-30250**	--		--
Class 3	59610	59737		59921		0.963		-29747	1		< .001
**Class 4**	**58053**	**58212**		**58444**		**0.957**		**-28953**	1		< .001
Class 5	57603	57795		58075		0.943		-28714	1		< .001
Class 6	57295	57520		57847		0.928		-28544	1		< .001
Solution based on 2 classes	Class counts and proportions for the latent classes				Average latent class probabilities for most likely latent class membership (row) by latent class (column)						
	Counts		Proportions		Class 1		Class 2				
Class 1	164		.10		.92		.08				
Class 2	1411		.90		.01		.99				
Solution based on 4 classes	Class counts and proportions for the latent classes				Average latent class probabilities for most likely latent class membership (row) by latent class (column)						
	Counts		Proportions		Class 1		Class 2		Class 3	Class 4	
Class 1	112		.07		.94		.04		.02	.00	
Class 2	677		.43		.00		.99		.01	.00	
Class 3	623		.40		.00		.03		.97	.00	
Class 4	163		.10		.00		.00		.00	1	

The entropy value, however, was greatest for the 3-class solution but only marginally higher than the 2-, 4-, and 5-class solutions (3-class = .963, 2-class = .952, 4-class = .957, 5-class = .943). Thus, we turned our attention to the proportion of class membership and the interpretability of each class. The 5-class solution had a single class comprising 2% of the sample (i.e., class 1), thus, was a sub-optimal solution. The 2-, 3-, and 4-class solutions had adequate proportions of members in each class and the average latent class probabilities for the most likely latent class membership were all greater than 90% for the assigned class (see lower section of Table 2: high values on the diagonal and low values off the diagonal indicate goodness of classification).

Based on interpretation of the 2-, 3-, and 4-class solutions (see Fig 1), there are two distinct profiles: those who are compliant (pictured by a blue line in the 2-class solution, and blue, green, and yellow lines in the 4-class solution) and those who are non-compliant with COVID-19 protective measures (pictured by a red line for both the 2-class and 4-class solution). The 3-class solution offered little beyond the interpretation of the 2-class solution. However, the 4-class solution provided further insight into the characteristics of the large compliant group based on demographics and health risk factors (see next section for class interpretation). Thus, the 2- and 4-class solutions appear to be reasonable representations of the profiles.

#### Interpretation of the 4-class solution

The percentage of participants in each of the 4 classes were: 7% in Class 1 (n = 112), 43% in Class 2 (n = 677), 40% in Class 3 (n = 623), and 10% in Class 4 (n = 163). It should be pointed out that the number of people in Class 1, the non-compliant group, is comparatively small. However, given the reality of the COVID-19 pandemic, even a small non-compliant group of people can be a major influence on the spread of the virus (e.g., super-spreaders). Moreover, there were likely varying degrees of non-compliance behaviours distributed amongst the other three classes, and borderline instances were classified as non-compliant in the forthcoming 2-class model presented below, where 10% of people comprised the non-compliant group.

Fig 1A and 1B present mean scores on the 14 variables included in LPA. Demographic and health characteristics for each Class are summarised in Table 3. Mean levels on the demographic variables were distinct for the four classes. Class 1 had the lowest mean scores on the measures related to COVID-19, including avoidant, preventive, and management of illness behaviours, self-reported compliance, worry about COVID-19, perceived benefits, response efficacy, and perceived barriers beliefs (reverse-coded for ease of graphical representation). Thus, this group appeared to be non-compliant with recommendations. Classes 2, 3, and 4 did not differ from each other on the COVID-19 measures and appeared to be compliant with recommendations. For prosocial behaviours, Class 1 was slightly higher than Class 2 but did not differ from Classes 3 or 4. There were no other differences between classes on pro- and anti-social behaviours. Class 1 members are relatively young individuals (M = 29.1, SD = 12.8), currently with relatively low levels of education (average highest attainment being vocational/trade certificate), good physical health, and no pre-existing health conditions.

**Table 3:** Mean demographic and health information for each class.

Class	Age	Education	Health conditions	Physical health
1	29.11 (12.79)	4.23 (1.49)	0.02 (0.13)	3.01 (0.62)
2	25.25 (11.10)	3.11 (0.34)	0.00 (0.00)	2.99 (0.72)
3	33.84 (12.11)	6.26 (0.71)	0.00 (0.00)	3.00 (0.64)
4	39.33 (16.25)	5.03 (1.66)	1.23 (0.50)	2.40 (0.75)

Similar to Class 1, members of Class 2 are young, and indeed the youngest of the sample (M = 25.2, SD = 11.1), with good physical health, no pre-existing health conditions, and majority currently at university (54% in this group were recruited from the University pool; majority [87%] reported high school as their highest educational attainment). Dissimilar to Class 1, this group of young people scored higher on COVID-19 measures, indicating compliance.

Classes 3 and 4 were also compliant with protective behaviours. Class 3 were approaching middle age (M = 33.8, SD = 12.1), had the highest level of education (average highest attainment being bachelor’s degree), good physical health, and no pre-existing health conditions. Class 4 were also middle aged (M = 39.3, SD = 16.2), had the second highest level of education (average highest attainment about associate degree), but reported impaired physical health and pre-existing health conditions. We interpreted each class based on these within-class variations: Class 1 as “*Non-compliant*”; Class 2 as “*Young and compliant”;* Class 3 as “*Educated and compliant”*; and Class 4 as “*Vulnerable health and compliant”*.

Given the main differences between the three compliant groups were found in age, education and health, for parsimony, all further analyses were performed on the classes extracted in the 2-class solution (*Compliant* and *Non-compliant*), which included a larger proportion of participants (10% rather than 7%) in Class 1 (non-compliant). This increase is due to the improved classification of borderline cases within the three compliant groups.

#### Country membership and gender in the 2-class solution

The relationships between the latent classes, country of residence, and gender were examined via the proportion of participants belonging to each class (see Table 4). The majority of participants were compliant (Class 2), with the proportion ranging from 82% in the US to 94% in Canada and UK, and 86% for male and 92% for female. A smaller proportion of individuals were non-compliant (Class 1), with membership ranging from 6% for Canada and UK to 18% for the US, and 14% for males and 8% for females. The US and Australia had a larger proportion of members in this class compared to Canada and the UK. There were a greater proportion of males in the *“Non-compliant”* class compared to females. Chi-square tests indicated that latent class had a significant association with country of residence (*X*^*2*^_3, N = 1575_ = 31.48, *p* < .001) and gender (*X*^*2*^_1, N = 1575_ = 13.21, *p* < .01).

**Table 4:** Proportion of individuals in LPA classes from each country and gender (N = 1575).

Class	Australia	Canada	UK	US	Male	Female	Total
1 Non-compliant	.12	.06	.06	.18	.14	.08	.10
2 Compliant	.88	.94	.94	.82	.86	.92	.90

#### Differences between non-compliant and compliant classes in the 2-class solution

Independent samples *t*-tests were conducted to examine differences between classes on psychological, behavioural, and cultural variables.

*Psychological variables*. Compared to compliant individuals, non-compliant individuals were significantly lower on Agreeableness, Intellect, and three coping strategies adaptive for COVID-19 (self-distraction, active coping, planning), and significantly higher on Extraversion, and three maladaptive coping strategies (denial, substance use, behavioural disengagement; Table 5). It should be noted that self-distraction (being involved in other activities e.g., work, watching television, etc.) was originally proposed to be a maladaptive coping strategy. However, during the lockdown period of the pandemic, this coping strategy was arguably an adaptive coping mechanism, and we will discuss it as such. No other significant differences were found.

**Table 5:** Independent samples t-tests on the difference between classes on psychological variables.

Measure	IC	Mean Overall (SD)	Mean Non-compliant (SD)	Mean Compliant (SD)	df	*t*	*p*	*d*	95% CI	
									Low	High
*Personality*										
**Agreeableness**	**.78**	**15.62 (3.13)**	**14.73 (3.01)**	**15.73 (3.13)**	**1573**	**-3.88**	**< .001**	**0.32**	**-1.50**	**-0.49**
Conscientiousness	.66	13.81 (3.20)	13.62 (2.94)	13.83 (3.22)	1573	-0.81	.42	-	-0.73	0.30
**Extraversion**	**.85**	**11.18 (4.09)**	**12.14 (3.68)**	**11.07 (4.12)**	**1573**	**3.16**	**< .001**	**0.26**	**0.40**	**1.72**
**Intellect**	**.72**	**15.13 (3.06)**	**14.67 (2.82)**	**15.18 (3.09)**	**1573**	**-2.01**	**.04**	**0.17**	**-1.00**	**-0.01**
Neuroticism	.78	11.82 (3.63)	11.54 (3.75)	11.85 (3.62)	1573	-1.04	.30	-	-0.90	0.28
*Cognitive Abilities*										
CRT accuracy	.91	48.12 (33.55)	45.76 (36.74)	48.47 (33.10)	462	-0.58	.56	-	-11.90	6.49
Belief accuracy	.79	56.01 (29.92)	53.02 (30.47)	56.44 (29.86)	460	-0.81	.42	-	-11.68	4.84
EAT accuracy	.74	67.52 (19.44)	64.45 (20.85)	67.95 (19.21)	865	-1.74	.08	-	-7.46	0.44
Trait accuracy	.88	56.85 (23.33)	55.50 (26.32)	57.03 (22.92)	456	-0.46	.65	-	-8.08	5.01
*Resilience*, *Adaptability & Coping*										
Resilience	.88	25.76 (6.32)	26.01 (6.45)	25.73 (6.31)	1573	0.54	0.59	0.04	-0.74	1.30
Adaptability	.88	25.57 (4.02)	25.59 (3.96)	25.57 (4.03)	1573	0.04	0.97	0.00	-0.64	0.66
**Active coping**	**.64**	**5.02 (1.57)**	**4.73 (1.59)**	**5.05 (1.56)**	**1573**	**-2.47**	**.01**	**0.20**	**-0.57**	**-0.07**
**Behavioural disengagement**	**.71**	**2.94 (1.31)**	**3.22 (1.46)**	**2.91 (1.29)**	**1573**	**2.87**	**< .001**	**0.24**	**0.10**	**0.52**
**Denial**	**.72**	**2.54 (1.08)**	**2.82 (1.41)**	**2.50 (1.03)**	**1573**	**3.59**	**< .001**	**0.30**	**0.14**	**0.49**
Emotional support	.81	4.39 (1.75)	4.19 (1.84)	4.41 (1.74)	1573	-1.50	.13	-	-0.50	0.07
**Humour**	**.85**	**4.37 (1.85)**	**5.00 (1.89)**	**4.29 (1.84)**	**1573**	**4.68**	**< .001**	**0.39**	**0.41**	**1.01**
Instrumental Support	.80	4.02 (1.61)	3.80 (1.61)	4.04 (1.61)	1573	-1.85	.06	-	-0.51	0.01
**Planning**	**.68**	**4.84 (1.60)**	**4.38 (1.63)**	**4.89 (1.59)**	**1573**	**-3.86**	**< .001**	**0.32**	**-0.77**	**-0.25**
Positive reframing	.77	5.04 (1.68)	4.94 (1.7)	5.05 (1.67)	1573	-0.83	.41	-	-0.39	0.16
**Religion**	**.87**	3.17 (1.73)	3.42 (1.78)	3.14 (1.72)	1573	1.91	.06	-	-0.01	0.55
**Self-blame**	**.58**	3.08 (1.33)	3.18 (1.49)	3.07 (1.31)	1573	0.99	.32	-	-0.11	0.32
**Self-distraction**	**.41**	**5.89 (1.54)**	**5.32 (1.68)**	**5.95 (1.51)**	**1573**	**-5.01**	**< .001**	**0.41**	**-0.88**	**-0.39**
**Substance Use**	**.96**	**2.85 (1.47)**	**3.19 (1.70)**	**2.81 (1.44)**	**1573**	**3.10**	**< .001**	**0.26**	**0.14**	**0.61**
Venting	.61	3.70 (1.40)	3.77 (1.52)	3.69 (1.38)	1573	0.72	.47	-	-0.14	0.31

Note: IC = internal consistency estimate computed using Cronbach’s Alpha (α); *d* = Cohen’s d.

We also conducted a series of between-subject ANOVAs with post-hoc contrasts (Tukey HSD procedure) to examine cross-cultural differences on the coping strategies. Due to very small effect sizes (η^2^ ranged between .01 for denial, humour, planning, substance use and venting to .03 for religion), these results are briefly summarised here but presented in full in Appendix B of the S1 Appendix. That is, compared with UK participants, Australians reported significantly higher levels of adaptive coping strategies (humour and religion) and lower levels of maladaptive coping strategies (denial and substance use). Compared with UK participants, Americans also reported significantly higher levels of adaptive coping strategies (planning and religion) and higher levels of a maladaptive coping strategy (venting). Compared with US participants, Australians reported significantly lower levels of adaptive coping strategies (planning and religion). Australians, however, reported significantly lower levels of denial (maladaptive coping strategy) than participants from Canada. UK participants reported significantly lower levels of religion (adaptive coping strategy) than participants from Canada.

*Information consumption*. Compared with the compliant class, the non-compliant class checked the news and the legitimacy of sources significantly less, used fewer official sources (e.g., government and health authority websites) for COVID-19 information, and had lower levels of trust in information sources (Table 6).

**Table 6:** Independent samples t-tests on the difference between classes on information consumption (df = 1573).

Measure	Mean Overall (SD)	Mean Non-compliant (SD)	Mean Compliant (SD)	*t*	*p*	*d*	95% CI	
							Low	High
**Check news**	**3.68 (1.03)**	**3.27 (1.10)**	**3.72 (1.01)**	**-5.33**	**< .001**	**0.44**	**-0.61**	**-0.28**
**Official sources**	**0.00 (1.00)**	**-0.34 (0.99)**	**0.04 (0.99)**	**-4.57**	**< .001**	**0.38**	**-0.54**	**-0.21**
Casual sources	0.00 (1.00)	-0.10 (1.03)	0.01 (1.00)	-1.29	.20	-	-0.27	0.06
**Source check**	**3.41 (1.11)**	**3.16 (1.01)**	**3.44 (1.11)**	**-3.02**	**< .001**	**0.25**	**-0.45**	**-0.10**
**Official trust**	**0.00 (1.00)**	**-0.59 (1.22)**	**0.07 (0.95)**	**-8.18**	**< .001**	**0.67**	**-0.82**	**-0.50**
**Casual trust**	**0.00 (1.00)**	**-0.19 (0.95)**	**0.02 (1.00)**	**-2.59**	**.01**	**0.21**	**-0.38**	**-0.05**

Note: *d* = Cohen’s d. Given measurement restrictions of these variable, IC estimates were not estimated.

*Political and cultural variables*. The non-compliant group was significantly lower than the compliant group on ratings of government truthfulness and cultural tightness-looseness but were higher on reactance and amorality (Table 7). A Mann-Whitney test indicated the two classes differed in their rating of the government’s response from insufficient to too extreme (W = 49784, *p <* .001). None of the other variables differed between the classes.

**Table 7:** Series of t-tests on the difference between classes on political and cultural variables (df = 1573 for all variables but amorality [df = 409]).

Measure	IC	Mean Overall (SD)	Mean Non-compliant (SD)	Mean Compliant (SD)	*t*	*p*	*d*	95% CI	
								Low	High
**Government truthfulness**	**-**	**3.38 (1.24)**	**3.14 (1.10)**	**3.41 (1.24)**	**-2.63**	**.01**	**0.22**	**-0.46**	**-0.07**
Government satisfaction	-	3.15 (1.19)	3.09 (1.04)	3.16 (1.20)	-0.72	.47	-	-0.26	0.12
Conservatism	.55	9.71 (2.44)	9.88 (2.17)	9.69 (2.47)	0.96	.34	-	-0.20	0.59
**Reactance**	**.86**	**40.07 (8.30)**	**43.54 (7.75)**	**39.66 (8.27)**	**5.72**	**< .001**	**0.47**	**2.55**	**5.21**
**Cultural tight/loose**	**.68**	**23.70 (3.70)**	**22.86 (3.46)**	**23.79 (3.72)**	**-3.05**	**< .001**	**0.25**	**-1.53**	**-0.33**
RWA	.65	9.04 (2.30)	8.96 (2.19)	9.05 (2.32)	-0.47	.64	-	-0.46	0.28
**Amorality**	**.64**	**13.90 (3.42)**	**14.98 (3.85)**	**13.75 (3.34)**	**2.39**	**.02**	**0.36**	**0.22**	**2.24**

Note: IC = internal consistency estimate which was computed using Cronbach’s Alpha (*α*) for all variables; *d* = Cohen’s d; RWA = right-wing authoritarianism.

*Future behaviours*. Non-compliant individuals anticipated leaving home significantly more frequently than compliant individuals for the following reasons: 1. Going to work, 2. Physical activity, 3. To get food, 4. To care for dependents, 5. To meet friends or family, 6. Religious reasons, 7. Because they are bored, and 8. To exercise their right to freedom (Table 8). While the first four reasons seem to be motivated by living and caring necessities, and, to various degrees, were generally permitted in the four countries examined, the last four reasons were not considered essential reasons to leave home during the pandemic.

**Table 8:** Series of t-tests on the difference between classes on future behaviours (df = 938).

Measure	Mean Overall (SD)	Mean Non-compliant (SD)	Mean Compliant (SD)	*t*	*p*	*d*	95% CI	
							Low High	
**Going to work**	**4.21 (1.18)**	**3.68 (1.34)**	**4.29 (1.14)**	**5.30**	**< .001**	**0.52**	**0.38**	**0.83**
Walking pet	4.30 (1.25)	4.4 (1.14)	4.28 (1.26)	-1.00	.32	-	-0.36	0.12
**Physical activity**	**3.28 (1.21)**	**3.06 (1.14)**	**3.31 (1.21)**	**2.12**	**.03**	**0.21**	**0.02**	**0.48**
**Get food**	**3.58 (0.75)**	**3.36 (0.83)**	**3.61 (0.73)**	**3.37**	**< .001**	**0.33**	**0.10**	**0.39**
Pharmacy	4.67 (0.58)	4.61 (0.69)	4.68 (0.56)	1.22	.22	-	-0.04	0.18
Medical treatment	4.92 (0.35)	4.92 (0.39)	4.92 (0.34)	-0.17	.86	-	-0.07	0.06
**Care dependents**	**4.76 (0.70)**	**4.61 (0.83)**	**4.78 (0.68)**	**2.55**	**.01**	**0.25**	**0.04**	**0.31**
**Meet friends/family**	**4.67 (0.65)**	**4.13 (0.91)**	**4.75 (0.56)**	**10.32**	**< .001**	**1.03**	**0.51**	**0.74**
**Religion**	**4.97 (0.29)**	**4.86 (0.56)**	**4.98 (0.22)**	**4.50**	**< .001**	**0.46**	**0.07**	**0.18**
**Bored**	**4.08 (1.14)**	**3.47 (1.23)**	**4.17 (1.1)**	**6.36**	**< .001**	**0.63**	**0.48**	**0.91**
**Right to freedom**	**4.55 (0.93)**	**3.82 (1.27)**	**4.66 (0.82)**	**9.62**	**< .001**	**0.96**	**0.67**	**1.01**

Note: *d* = Cohen’s d.

Particularly pronounced was the difference in intention to meet friends or family, which, at the time of data collection was generally either recommended or mandated against in all four countries.

## Discussion

The current study is the first to provide a holistic view of the factors influencing behavioural compliance with protective measures during COVID-19. In doing so, we integrated an extensive battery of constructs based on theories from multiple paradigms, including epidemiology, health, differential, and cultural psychology, revealing a complex picture of behaviour and the need for targeted interventions. The novel person-centred approach offers insight into different clusters/groups within the population based on behaviours, attitudes, and key demographics. The sample clustered into two broad groups: those compliant and those not. Whilst the majority fell into the compliant group (90%); 10% of individuals reported non-compliant behaviours and attitudes, which is enough to be cause for concern given the risk of exponential spread.

The compliant and non-compliant groups differed on a number of variables, including beliefs about protective measures, social attitudes, and personality. There was remarkable consistency across the four countries surveyed; and surprisingly, the non-compliant group was not populated simply by young people. The non-compliant group was the second youngest amongst four identified sub-groups, however, and the youngest individuals formed a distinct cluster within the three compliant groups. In the two-class solution, compliant and non-compliant groups did not differ in age. This finding runs contrary to the oft-promoted media stereotype of the young, COVID-indifferent partygoers neglecting restrictions. The picture we discovered was much more complex and key take-away findings are discussed below.

### Compliance rates

Overall, we found a promisingly high (90%) rate of compliance, and each of the four countries displayed relatively similar compliance rates. However, we note that this sample was drawn early in the pandemic. These rates of compliance may change as the pandemic prolongs and people become more complacent. Further, country-level differences are likely to become more pronounced given the major differences between countries’ trajectories and regulations since the first wave.

### Differences in demographics and attitudes towards protective measures

The compliant group endorsed protective measures as beneficial and effective in leading to better health-related outcomes. By contrast, the non-compliant group appeared concerned with the social and economic cost of such measures. These findings align with health behaviour frameworks which propose that behaviour change is, in part, motivated by perceptions about the efficacy, benefits, and costs of behaviours [13, 14]. Compliant and non-compliant groups differed in their level of worry about COVID-19, consistent with pre-COVID and emerging COVID-19 research showing worry or fear is an important driver of positive behaviour change [2, 3, 6, 8, 12]. These findings support the Health Belief Model and Protection Motivation Theory which suggest that perceptions of severity of the threat, vulnerability to infection, efficacy of protective behaviours, self-efficacy, and perceived benefits and barriers of protective actions are the key beliefs driving health behaviour change and compliance [13, 14]. Although based on correlational data, these results stress the importance of targeting these perceptions to increase compliance.

Notably, on average, the groups did not differ on age, education, or physical health, nor were there differences in pro- or anti-social behaviours. Consistent with previous research, females showed higher rates of compliance than males [15].

The four-class solution allowed for a more nuanced view of the large compliant group, which split into three distinct groups, offering insight into possible motivations behind compliance. The largest group (Class 2) were the youngest, and largely university students. The second largest group (Class 3) were middle-aged and more highly educated; and a minority fit into a third compliant class (Class 4), who were older and had poorer physical health. To our knowledge, although intuitive, no previous study has demonstrated the existence of these classifications within the compliant population.

### Personality differentiates compliant and non-compliant groups

Unsurprisingly, the non-compliant group were more extraverted. This group indicated their plans to visit family and friends in the forthcoming week, characteristically extraverted behaviours. However, such behaviours are especially worrisome at the time of pandemic. Although it is difficult to change a psychological trait, to increase compliance, the self-centredness of certain manifestations of trait extraversion may need to be targeted, as they present a health risk factor to others.

Consistent with some emerging COVID-19 findings, the compliant group scored higher on intellect/openness and agreeableness [18, 19]. Contrary to what other COVID-19 research suggests [19, 53] we did not find any differences in conscientiousness or neuroticism between groups. Similarly, perceptions of being resilient and adaptable did not promote compliance during the first wave. This, however, may be the result of sampling during the first few months of the pandemic and the results may change with prolonged exposure to restrictions as the pandemic continues.

Finally, the compliant group were more likely to cope adaptively by self-distraction, planning, and using active strategies; whilst non-compliant people were more likely to cope through denial, substance use, and behavioural disengagement. This research was correlational; thus, no causal mechanism is implied. Instead, we suggest that future studies, should examine whether the promotion and acceptance of more adaptive strategies will lead to better management of isolation and boredom, and help to increase and maintain compliance. If this is the case, intervention strategies should include promotion and education of adaptive strategies, which might be disseminated through mainstream and social media in engaging ways.

### Information consumption

The compliant group reported greater use of official government and health information sources than the non-compliant group, suggesting compliant people are better-informed about COVID-19. Also supporting this notion, non-compliant individuals tend to check the legitimacy of sources less than compliant individuals. The groups did not differ in their use of casual information sources (e.g., social media, conversations), highlighting the potential for utilising casual sources for the dissemination of official information. The compliant group checked the news more frequently and expressed greater trust in all information sources than the non-compliant group. Research from the Avian flu pandemic showed that trust in both formal and informal information sources was associated with greater worry, and trust in formal information was linked to greater perceived effectiveness of hygiene behaviours [4]. It is possible that more frequent news-checking has similar impacts on worry and perceptions of protective behaviours, thus promoting compliance. Future studies should determine whether the dissemination of *official* and *reliable* information in accessible form (e.g., memes, short messages and videos) via a variety of news outlets, including casual (e.g., social media), may increase rates of compliance in the non-compliant group. However, this would require people to accurately evaluate the legitimacy of information to distinguish between official information and that which is not credible. Thus, targeted interventions focusing on education about how to check the credibility of information, would be of critical importance to foster greater recognition of fake and misleading news.

### Attitudes towards government and other cultural factors

Compliant individuals perceived their government as being more truthful than those non-compliant, though there were no differences in reports of satisfaction with their government’s response. Contradictorily, groups differed in their responses when asked whether they thought their government’s reaction to the COVID-19 outbreak was appropriate, too extreme, or insufficient; such that the compliant group was more likely to perceive their government’s reaction to be insufficient compared to the non-compliant group. This aligns with Fetzer et al.’s [34] finding that perceiving the government’s response to be insufficient is associated with greater worry about COVID-19, which in turn may motivate compliance. Conversely, the non-compliant group scored higher on reactance, indicating they are more likely to perceive rules as a threat to their freedom and thus resist them. Consistently, the non-compliant group reported looser cultural norms and higher amorality. A ‘loose’ culture is characterised by valuing freedom, hence is less accustomed to strict social norms such as those imposed during the pandemic. However, we note that the countries sampled are relatively culturally similar. High scores on amorality indicate disregard for moral values within society and are associated with self-interested behavioural choices that ignore COVID-19 guidelines. Whilst no causality is implied, it is possible that emphasising the message of common goals and moral responsibilities at the time of a global health crisis may foster higher compliance rates. Future studies should examine the most efficient messaging to target self-interests, reactance, and perceptions of looser cultural norms.

### Implications and future directions

Heterogeneity in the population poses a challenge to implementing widespread behaviour change policies. These strategies should be targeted for different profiles of individuals and focus on increasing the perceived benefits and efficacy of protective measures, reducing barriers, and fostering a functional level of worry. Several directions for future studies have already been proposed. The section below covers further implications of our findings.

Non-compliant individuals appear to distrust and be sceptical of both formal and informal information sources. Further research is needed to identify sources considered trustworthy by this group in order to optimise communication of health advice.

Maintaining compliance as restrictions remain in place for a prolonged period is critically important. Perceptions of being resilient and adaptable did not promote compliance during the first wave in our overall sample. However, it would be fruitful to examine resilience and adaptability beyond the first wave, under the threat of future waves and lockdowns. Further, perceptions of one’s resilience may change as the pandemic prolongs. Some might succumb to the challenges of the pandemic experience, whilst others may discover their strength and experience resilient growth, with both changes having profound effects on mental health.

Lastly, this study was conducted in the early stages of the pandemic. Mobile tracking data from several European countries suggests that people stayed home substantially less during the second wave from late 2020 to early 2021 than they did during the first wave [54]. Thus, further research is needed to examine whether the same profiles and predictors of behaviours emerge in these later stages when rates of compliance and behaviour change have fluctuated.

### Strengths and limitations

As a strength, this study addresses the intention-behaviour gap issue prominent in health behaviour research. Our measure of compliance captured behaviours within a critical period rather than asking participants to recall past behaviours. Equally important, we captured a comprehensive range of constructs, providing a holistic view of factors drawn from different psychology paradigms, which extends upon current research by integrating person- and variable-centred approaches to profile and examine characteristics of individuals. These findings may be used to inform strategies for improving and maintaining behaviour change.

We collected data from a large and diverse sample of people in four countries, giving good power and generalisability within that sample. However, a small proportion (18.4%) were collected using snowball recruitment, and using a university student pool (26.3%), contributing to selection bias. We also acknowledge that the interpretation is limited by the fact we sampled ‘WEIRD’ (Western, educated, industrialised, rich, democratic) countries. Though emerging research from other countries has reported findings consistent with ours. For example, distrust in government authorities was associated with non-compliance in Swiss [55], Nigerian [56], Italian, and French samples [57]. Additionally, males and those with less moral values showed lower compliance in Swiss adults [55], as did those with no worry about COVID-19 in Italian and French samples [57]. Further, perceived benefits and efficacy of protective measures have emerged as strong predictors of compliance across samples from a range of countries including Switzerland [58], Ethiopia [59], and China [60]. Nevertheless, we managed to capture a relatively heterogeneous sample, varying in age, gender, levels of education, pre-existing health conditions, and economic situation.

We also used brief versions of several scales and their psychometric properties may limit reliability. However, with few exceptions, most measures had reasonable to excellent reliability estimates. Newly developed measures showed promising preliminary psychometric properties but require further validation.

Moreover, while we captured different levels of education this research did not capture any other socio-economic status (SES) metrics. The emerging results indicate that in the USA, higher SES was related to earlier incidences of COVID-19 cases, but as regulations of social distancing were imposed, the growth of incidents was slower in higher SES countries with lower case fatality rates [61]. Future research needs to determine economic and social conditions that may have disadvantaged different populations as the pandemic progressed within the four countries examined in this research and across the globe. For instance, density of living situations, and reduced capacity to access healthcare, reliable information, and to work from home are important barriers to overall compliance behaviours.

Finally, during the time of data collection, with some caveats, the four countries sampled had employed similar approaches to controlling the spread of COVID-19. This likely contributed to consistency between the four countries surveyed in the profiles identified and their characteristics. Although the consistency in personal characteristics and behavioural patterns across four countries is encouraging, this finding needs to be replicated and extended as the rules and conditions change in these four countries.

### Overall conclusion

Compliance with protective behaviours is paramount in containing the COVID-19 pandemic and allowing people to return to their everyday activities under the new ‘COVID normal’. This research is the first to adopt a person-centred approach, whilst capturing a comprehensive suite of constructs from multiple paradigms within psychology and beyond. This allowed us to clarify the complex nature of compliant and non-compliant behaviours. By promoting greater appreciation of the complexity of behaviour during COVID-19, this research provides a critical platform to inform future studies, public health policy, and targeted behaviour change interventions during pandemics.

The non-compliant group expressed greater agreement with statements that protective measures are costly and inconvenient than the compliant group, and were less worried about COVID-19 than the compliant group. Further, the non-compliant group was less agreeable, more extraverted, lower on intellect/openness, and more likely to cope with COVID-19 through denial, substance use, and behavioural disengagement. They also checked the news and official sources for COVID-19 information less frequently, were less likely to verify the accuracy of information, and reported less trust in information sources and the government. The non-compliant group scored higher on reactance—indicating they are more motivated to fight for their individual freedom; perceived their culture as looser—indicating perceptions that individual freedom is of high value; and scored higher on amorality—indicating greater self-interest and disregard for social obligations than the compliant group. Alarmingly, the non-compliant group were more likely than the compliant group to leave their home in the following week to meet friends/family, for religious reasons, because they are bored, and to exercise their right to freedom. These differences highlight a number of important characteristics of non-compliers which could be targeted to improve compliance. For instance, the compliant group used more adaptive coping strategies including distraction, active coping, and planning. Public education and the promotion of adaptive coping strategies may thus help to enhance compliance with protective measures. Further, these findings highlight the importance of regulating and monitoring misinformation as non-compliers appear not to use official sources for COVID-19 information, nor do they tend to verify the legitimacy of information. Hence, disseminating official information through a variety of casual sources might reach a larger audience; however, education is needed about how to evaluate the credibility information. Lastly, framing public health messages to appeal to self-interests may also be more effective in promoting positive behaviour change amongst non-compliant people than appealing to social obligations and the need to protect others.

## Supporting information

S1 Appendix Newly developed COVID-19 related measures: Items and scoring. (DOCX)

## References

[1] World Health Organization [Internet]. Timeline of WHO’s response to COVID-19. [cited 2020 Aug 3]. Available from: https://www.who.int/news-room/detail/29-06-2020-covidtimeline

[2] Leung GM, Quah S, Ho LM, Ho SY, Hedley AJ, Lee HP, et al. A tale of two cities: Community psychobehavioral surveillance in Hong Kong and Singapore during the severe acute respiratory syndrome epidemic. Infect Control Hosp Epidemiol. 2004;25(12): 1033–1041. doi: 10.1086/502340 15636289

[3] Leung GM, Ho LM, Chan SKK, Ho S, Bacon-Shone J, Choy R, et al. Longitudinal assessment of community psycho-behavioural responses during and after the 2003 outbreak of severe acute respiratory syndrome in Hong Kong. Clin Infect Dis. 2005;40(12): 1713–1720. doi: 10.1086/429923 15909256

[4] Liao Q, Cowling BJ, Lam WWT, Fielding R. The influence of social-cognitive factors on personal hygiene practices to protect against influenzas: Using modelling to compare Avian A/H5N1 and 2009 pandemic A/H1N1 influenzas in Hong Kong. Int J Behav Med. 2011;18(2): 93–104. doi: 10.1007/s12529-010-9123-8 20949342 PMC3088805

[5] Bish A, Michie S. Demographic and attitudinal determinants of protective behaviours during a pandemic: a review. Br J Health Psychol. 2010;15(4): 797–824. doi: 10.1348/135910710X485826 20109274 PMC7185452

[6] Barber SJ, Kim H. COVID-19 Worries and Behavior Changes in Older and Younger Men and Women. J Gerontol B Psychol Sci Soc Sci. 2021;18(76): e17–e23. doi: 10.1093/geronb/gbaa068 32427341 PMC7313781

[7] Clark C, Davila A, Regis M, Kraus S. Predictors of COVID-19 voluntary compliance behaviors: An international investigation. Glob Transit. 2020;2: 76–82. doi: 10.1016/j.glt.2020.06.003 32835202 PMC7318969

[8] Harper CA, Satchell LP, Fido D, Latzman RD. Functional Fear Predicts Public Health Compliance in the COVID-19 Pandemic. Int J Ment Health Addict. 2020. doi: 10.1007/s11469-020-00281-5 32346359 PMC7185265

[9] Seale H, Heywood AE, Leask J, Sheel M, Thomas S, Durrheim DN, et al. COVID-19 is rapidly changing: Examining public perceptions and behaviors in response to this evolving pandemic. PLoS One. 2020;15(6): e0235112. doi: 10.1371/journal.pone.0235112 32574184 PMC7310732

[10] Xie W, Campbell S, Zhang W. (2020). Working memory capacity predicts individual differences in social-distancing compliance during the COVID-19 pandemic in the United States. Proc Natl Acad Sci U S A. 2020;117(30): 17667–17674. doi: 10.1073/pnas.2008868117 32651280 PMC7395511

[11] Petersen KJ, Qualter P, Humphrey N. The application of latent class analysis for investigating population child mental health: A systematic review. Front Psychol. 2019. doi: 10.3389/fpsyg.2019.01214 31191405 PMC6548989

[12] Brug J, Aro AR, Oenema A, de Zwart O, Richardus JH, & Bishop GD. SARS risk perception, knowledge, precautions, and information sources, the Netherlands. Emerg Infect Dis. 2004;10(8): 1486–1489. doi: 10.3201/eid1008.040283 15496256 PMC3320399

[13] Rogers RW. A protection motivation theory of fear appeals and attitude change. J Psychol. 1975;91(1): 93–114. doi: 10.1080/00223980.1975.9915803 28136248

[14] Carpenter CJ. A meta-analysis of the effectiveness of Health Belief Model variables in predicting behavior. Health Commun. 2010;25(8): 661–669. doi: 10.1080/10410236.2010.521906 21153982

[15] Moran KM, Del Valle SY. A meta-analysis of the association between gender and protective behaviors in response to respiratory epidemics and pandemics. PLOS One, 2016;11(10): e016541. doi: 10.1371/journal.pone.0164541 27768704 PMC5074573

[16] John OP, Srivastava S. The big five trait taxonomy: History, measurement, and theoretical perspectives. In: Pervin LA, John OP, editors. Handbook of personality: Theory and research. Guildford Press; 1999. pp. 102–138.

[17] Bogg T, & Roberts BW. Conscientiousness and health-related behaviors: A meta-analysis of the leading behavioral contributors to mortality. Psychol Bull. 2004;130(6): 887–919. doi: 10.1037/0033-2909.130.6.887 15535742

[18] Aschwanden D, Strickhouser JE, Sesker AA, Lee JH, Luchetti M, Stephan Y, et al. Psychological and behavioural responses to coronavirus disease 2019: The role of personality. Eur J Pers. 2020; 35(1), 51–66. doi: 10.1002/per.2281 32836766 PMC7361622

[19] Blagov PS. Adaptive and dark personality in the COVID-19 pandemic: Predicting health-behaviour endorsement and the appeal of public-health messages. Soc Psychol Personal Sci. 2020. doi: 10.1177/1948550620936439 PMC7342937 38602980

[20] Frederick S. Cognitive reflection and decision making. J Econ Perspect. 2005; 19(4): 25–42. doi: 10.1257/089533005775196732

[21] Toplak ME, West RF, Stanovich KE. The Cognitive Reflection Test as a predictor of performance on heuristics-and-biases tasks. Mem Cognit. 2011;39(7): 1275–1289. doi: 10.3758/s13421-011-0104-1 21541821

[22] Marroquín B, Vine B, Morgan R. Mental health during the COVID-19 pandemic: Effects of stay-at-home policies, social distancing behavior, and social resources. Psych Res. 2020;293: 113419. doi: 10.1016/j.psychres.2020.113419 32861098 PMC7439968

[23] Serafini G, Parmigiani B, Amerio A, Aguglia A, Sher L, Amore M. The psychological impact of COVID-19 on the mental health in the general population. QJM. 2020;113(8), 531–537. doi: 10.1093/qjmed/hcaa201 32569360 PMC7337855

[24] Carver CS, Scheier MF, Weintraub JK. Assessing coping strategies: A theoretically based approach. J Pers Soc Psychol. 1989; 56(2): 267–283. doi: 10.1037//0022-3514.56.2.267 2926629

[25] Davydov DM, Stewart R, Ritchie K, & Chaudieu I. Resilience and mental health. Clin Psychol Rev. 2010;30: 479–495. doi: 10.1016/j.cpr.2010.03.003 20395025

[26] Ployhart RE, Bliese PD. Individual adaptability (I-ADAPT) theory: Conceptualizing the antecedents, consequences, and measurement of individual differences in adaptability. In: Pierce LG, Salas E, editors. Understanding Adaptability: A prerequisite for effective performance within complex environments. Amsterdam, Netherlands: Elsevier; 2006. pp. 3–39.

[27] Sibley CG, Greaves LM, Satherley N, Wilson MS, Overall NC, Lee CHJ, et al. Effects of the COVID-19 pandemic and nationwide lockdown on trust, attitudes toward government, and well-being. Am Psychol. 2020;75(5): 618–630. doi: 10.1037/amp0000662 32496074

[28] Stankov L. Conservative syndrome: Individual and cross-cultural differences. J Cross Cult Psychol, 2017;48(6): 1–11. doi: 10.1177/0022022117709984

[29] Saunders BA, & Ngo J. The right-wing authoritarianism scale. In: Zeigler-Hill V, Schackelford T, editors. Encyclopedia of personality and individual differences. Springer; 2017.

[30] Brehm JW. A theory of psychological reactance. Acad Press. 1966.

[31] Gelfand MJ, Raver JL, Nishii L, Leslie LA, Lun J, Lim BC, et al. Differences between tight and loose cultures: A 33-nation study. Science. 2011;332(6033): 1100–1104. doi: 10.1126/science.1197754 21617077

[32] Van Bavel JJ, Baicker K, Boggio PS, Capraro V, Cichocka A, Cikara M, et al. Using social and behavioural science to support COVID-19 pandemic response. Nat Hum Behav. 2020; 4: 460–471. doi: 10.1038/s41562-020-0884-z 32355299

[33] Basch CH, Hillyer GC, Meleo-Erwin Z, Mohlman J, Cosgrove A, Quinones N. News coverage of the COVID-19 pandemic: Missed opportunities to promote health sustaining behaviors. Infect Dis Health. 2020;25(3): 205–209. doi: 10.1016/j.idh.2020.05.001 32426559 PMC7229940

[34] Fetzer T, Witte M, Hensel L, Jachimowicz JM, Haushofer J, Ivchenko A, et al. Perceptions of an insufficient government response at the onset of the COVID-19 pandemic are associated with lower mental well-being. PsyArXiv [Preprint]. 2020 [posted 2020 April 16; revised 2021 March 25]. Available from: https://psyarxiv.com/3kfmh/.

[35] Donnellan MB, Oswald FL, Baird BM, Lucas RE. The mini-IPIP scales: Tiny-yet-effective measures of the Big Five factors of personality. Psychol Assess. 2006;18(2): 192–203. doi: 10.1037/1040-3590.18.2.192 16768595

[36] Stankov L. Gf–Gc quickie test battery. Unpublished test battery. University of Sydney; 1997.

[37] Jackson SA, Kleitman S. Individual differences in decision-making and confidence: Capturing decision tendencies in a fictitious medical test. Metacogn Learn. 2014;9: 25–49. doi: 10.1007/s11409-013-9110-y

[38] Jackson SA, Kleitman S, Howie P, Stankov L. Cognitive abilities, monitoring confidence, and thresholds explain individual differences in heuristics and biases. Front Psychol. 2016;7: 1559. doi: 10.3389/fpsyg.2016.01559 27790170 PMC5062089

[39] Toplak ME, West RF, Stanovich KE. Assessing miserly information processing: An expansion of the Cognitive Reflection Test. Think Reason. 2014;20(2): 147–168. doi: 10.1080/13546783.2013.844729

[40] Markovits H, Nantel G. The belief-bias effect in the production and evaluation of logical conclusions. Mem Cognit. 1989;17(1): 11–17. doi: 10.3758/bf03199552 2913452

[41] Sirota M, Juanchich M. Effect of response format on cognitive reflection: Validating a two- and four-option multiple choice question version of the Cognitive Reflection Test. Behav Res Methods. 2018;50: 2511–2522. doi: 10.3758/s13428-018-1029-4 29589333

[42] Campbell-Sills L, Stein MB. Psychometric analysis and refinement of the Connor-Davidson Resilience Scale (CD-RISC): Validation of a 10-item measure of resilience. J Trauma Stress. 2007;20(6): 1019–1028. doi: 10.1002/jts.20271 18157881

[43] Zhang LM, Aidman E, Burns B, Kleitman S. Integrating self-report and performance-based assessments of adaptability in a university context. J Res Pers. 2020; 88. doi: 10.1016/j.jrp.2020.103988

[44] Carver CS. You want to measure coping but your protocol’s too long: Consider the Brief COPE. Int J Behav Med. 1997;4(1): 92–100. doi: 10.1207/s15327558ijbm0401_6 16250744

[45] Everett JAC. The 12 Item Social and Economic Conservatism Scale (SECS). PLOS One. 2013;8(12). doi: 10.1371/journal.pone.0082131 24349200 PMC3859575

[46] Beierlein C, Asbrock F, Kauff M, Schmidt P. Die Kurzskala Autoritarismus (KSA-3). GESIS Working Papers: Leibniz-Institut für Sozialwissenschaften, Mannheim; 2014.

[47] Hong SM, Page S. A Psychological Reactance Scale: Development, Factor Structure and Reliability. Psychol Rep. 1989;64(3): 1323–1326. doi: 10.2466/pr0.1989.64.3c.1323

[48] Shen L, Dillard JP. Psychological properties of the Hong Psychological Reactance Scale. J Pers Assess. 2005;85(1): 74–81. doi: 10.1207/s15327752jpa8501_07 16083386

[49] Stankov L, Knezevic G. Amoral social attitudes and value systems among Serbs and Australians. Aust J Psychol. 2005;57(2): 115–128. doi: 10.1080/00049530500048649

[50] Tabachnick BG, Fidell LS. Using multivariate statistics. 5th ed. Allyn & Bacon/Pearson Education; 2007.

[51] Wang J, Wang X. Structural equation modelling: Methods and applications. Wiley; 2012.

[52] Brusco MJ, Shireman E, Steinley D. A comparison of latent class, K-means, and K-median methods for clustering dichotomous data. Psychol Methods. 2017;22(3):563–580. doi: 10.1037/met0000095 27607543 PMC5982597

[53] Abdelrahman M. Personality traits, risk perception, and protective behaviours of Arab residents of Qatar during the COVID-19 pandemic. Int J Ment Health Addict. 2020. doi: 10.1007/s11469-020-00352-7 32837433 PMC7307935

[54] Georganas S. Lockdown: mobile data suggests Europeans may not have followed rules as strictly in the second wave. The Conversation. 2021 March 22 [Cited 22 June 2021]. Available from: https://theconversation.com/lockdown-mobile-data-suggests-europeans-may-not-have-followed-rules-as-strictly-in-the-second-wave-154815.

[55] Nivette A, Ribeaud D, Murray A, Steinhoff A., Bechtiger L, Hepp U, et al. Non-compliance with COVID-19-related public health measures among young adults in Switzerland: Insights from a longitudinal cohort study. Soc Sci Med. 2021;268: 113370. doi: 10.1016/j.socscimed.2020.113370 32980677 PMC7493799

[56] Ezeibe CC, Ilo C, Ezeibe EN, Oguonu CN, Nwankwo NA, Ajaero CK, et al. Political distrust and the spread of COVID-19 in Nigeria. Glob Public Health. 2020;15(12): 1753–1766. doi: 10.1080/17441692.2020.1828987 33019916

[57] Lalot F, Heering MS, Rullo M, Travaglino GA, Abrams D. The dangers of distrustful complacency: Low concern and low political trust combine to undermine compliance with governmental restrictions in the emerging Covid-19 pandemic. Group Process Intergroup Relat. 2020. doi: 10.1177/1368430220967986

[58] Scholz U, Freund AM. Determinants of protective behaviours during a nationwide lockdown in the wake of the COVID-19 pandemic. British J Health Psyc. 2021. doi: 10.1111/bjhp.12513 33847029 PMC8250218

[59] Shewasinad Yehualashet S, Asefa KK, Mekonnen AG, Gemeda BN, Shiferaw WS, Aynalem YA, et al. Predictors of adherence to COVID-19 prevention measure among communities in North Shoa Zone, Ethiopia based on health belief model: A cross-sectional study. PLoS ONE. 2021;16(1): e0246006. doi: 10.1371/journal.pone.0246006 33481962 PMC7822535

[60] Dai B, Fu D, Meng G, Liu B, Li Q, Liu X. The effects of governmental and individual predictors on COVID-19 protective behaviors in China: a path analysis model. Public Adm Rev. 2020; doi: 10.1111/puar.13236 32836438 PMC7276878

[61] Clouston SAP, Natale G, Link BG. Socioeconomic inequalities in the spread of coronavirus-19 in the United States: A examination of the emergence of social inequalities. Soc Sci Med. 2021;268:113554. doi: 10.1016/j.socscimed.2020.113554 33308911 PMC7703549

