# Supplementary material for: To comply or not comply? A latent profile analysis of behaviours and attitudes during the COVID-19 pandemic
Source: PLoS One. 2021 Jul 29;16(7):e0255268. doi: 10.1371/journal.pone.0255268 (PMC8321369; doi:10.1371/journal.pone.0255268)
Supplement: S1 Appendix — (DOCX) [file pone.0255268.s001.docx]

**Appendix A: Newly Developed COVID-19 Related Measures: Items and scoring**

**Self-Report Compliance Measure**

Participants rated four items on a scale from 1 (strongly disagree) to 5 (strongly agree). A principal component analysis was run to determine dimensionality of the measure. Items and results are presented in Table A1. All items converged into a single dimension capturing 75.74% of variance. It was labelled *Self-Reported Compliance.* Cronbach’s alpha was .89, indicating high reliability.

Table A1. Items, Loadings, and Communalities for the Self-Report Compliance Measure.

| Item | Loading | *h*^2^ |
| --- | --- | --- |
| 1. I follow my government’s restrictions to protect myself from COVID-19 | .82 | .67 |
| 2. I follow my government’s restrictions to protect my friends and family from COVID-19 | .88 | .77 |
| 3. I follow my government’s restrictions to protect at-risk populations from COVID-19 | .89 | .78 |
| 4. I follow my government’s restrictions to protect people in general from COVID-19 | .90 | .81 |

*Note. h*^2^ = communalities

**Protective Behaviours Measure**

Participants were asked to rate the extent to which 12 items described their behaviour in the past week, on a sliding scale from 0 to 100. Items and their numbers are presented in Table A2. Items 1 to 4 were taken from Fetzer et al.’s (2020) past behaviour measure. Items 10 to 12 are modifications of Fetzer et al.’s item “If I had exhibited symptoms of sickness, I would have immediately informed the people around me”.

A principal components analysis (with Promax rotation) examined the dimensionality of this measure^[[1]](#footnote-1)^. The Kaiser criterion (eigenvalues greater than one) and scree plot indicated three dimensions, capturing 55.64% of variance. Items and results are presented in Table A2. The dimensions aligned with Bish and Michie’s (2010) categorisation of protective behaviours during a pandemic: *Avoidant Behaviours* (component 1), *Management of Illness Behaviours* (component 2), and *Preventive Behaviours* (component 3), and were labelled as such. Item 6 had cross-loadings on components 1 and 3, which is reasonable given that not shaking hands or hugging reflects both avoidance of others and personal hygiene. As component 3 was only defined by two other items, it was included with this subscale for scoring. Cronbach’s alphas were .78 (avoidant), .68 (management), and .56 (preventive). Estimates for Management and Preventive Behaviours were likely lower due to only containing three items; one of which in Preventive Behaviours had a lower loading on that component and cross-loaded.

Table A2. Items, Loadings, and Communalities for the Protective Behaviours Measure

| Item | 1 | 2 | 3 | h^2^ |
| --- | --- | --- | --- | --- |
| 8. I stopped meeting friends in person | **.82** | -.13 | .02 | .63 |
| 1. I stayed at home | **.76** | .10 | -.32 | .58 |
| 3. I kept a distance of at least two meters from other people | **.73** | .05 | .02 | .57 |
| 7. I avoided crowded places | **.69** | .16 | -.02 | .57 |
| 2. I did not attend social gatherings | **.58** | -.14 | .17 | .37 |
| 9. I stopped meeting at-risk people (e.g., elderly) | **.46** | .02 | .22 | .33 |
| 11. If I exhibited symptoms of sickness, I would have immediately called a doctor | -.14 | **.79** | .08 | .61 |
| 10. If I exhibited symptoms of sickness, I would have immediately informed friends/family | .00 | **.79** | .09 | .67 |
| 12. If I exhibited symptoms of sickness, I would have immediately self-isolated | .15 | **.78** | -.04 | .69 |
| 5. I coughed or sneezed into my elbow or a handkerchief | -.09 | .04 | **.82** | .65 |
| 4. I washed my hands more frequently | .06 | .14 | **.67** | .55 |
| 6. I stopped greeting people by shaking hands or hugging | **.44** | -.12 | **.44** | .45 |
| Component Correlations |  |  |  |  |
| 2 | .32 |  |  |  |
| 3 | .29 | .28 |  |  |

*Note.* Factor loadings over .30 are bolded; *h*^2^ = communalities

**Prosocial and ‘Self-Preservation’ Antisocial** **Behaviours Measure**

Participants were asked to rate the extent to which 8 items described their behaviour in the past week, on a sliding scale from 0 to 100. A principal component analysis (with Promax rotation) examined the dimensionality of this measure. The Kaiser criterion indicated three possible dimensions; however, one was only defined by two items, where three is the suggested minimum. The scree plot indicated two possible factors. The analysis was rerun constraining the solution to two factors for comparison. The two factors accounted for 44.60% of variance. This solution was retained given greater interpretability of dimensions, sufficient items on each dimension, and adequate loadings and communalities. Items, loadings, and communalities are presented in Table A3. Component 1 was labelled *Antisocial Behaviours* and Component 2 was labelled *Prosocial Behaviours.* Both dimensions had a low approaching .60 mark internal consistency estimate (Cronbach’s alpha) of .56, and shared a small correlation of .24.

Table A3. Items, Loadings, and Communalities of the Prosocial and ‘Self-Preservation’ Antisocial Behaviours Measure

| Item | 1 | 2 | *h*^2^ |
| --- | --- | --- | --- |
| 6. Bought more medical and sanitary products (e.g., masks, paracetamol) from the pharmacy than you normally would | **.86** | -.02 | .73 |
| 5. Bought more products (e.g., groceries from the supermarket than you normally would | **.85** | -.12 | .69 |
| 7. Had a verbal disagreement with friends/family/strangers about COVID-19 (directly or indirectly) | **.39** | .18 | .22 |
| 8. Had a physical altercation with friends/family/strangers about COVID-19 | **.38** | .24 | .25 |
| 4. Provided emotional support to strangers | .00 | **.74** | .54 |
| 2. Donated to businesses or individuals affected by COVID-19 | -.02 | **.66** | .42 |
| 3. Provided emotional support to your family and friends | -.02 | **.63** | .38 |
| 1. Delivered medical supplies, groceries or meals to at-risk populations (e.g., elderly) | .11 | **.55** | .34 |

*Note.* Factor loadings above .30 are bolded; *h*^2^ = communalities

**COVID-19 Beliefs Measure**

Participants rated the extent to which they agreed or disagreed with 10 items about COVID-19 protective measures, on a 5-point scale from 1 (strongly disagree) to 5 (strongly agree). Items are presented in Table A4. Items 1 to 3 were adapted from Fetzer et al. (2020). The original items were yes/no questions, and were modified into statements to fit the agree/disagree response scale. A principal component analysis examined the dimensionality of this measure. The Kaiser criterion and scree plot both indicated three components, capturing 57.46% of variance. The results are presented in Table A4. Component 1 was labelled *Perceived Benefits Beliefs*, defined by endorsement of strict protective measures that are regarded to be beneficial despite costs*.* Component 2 was labelled *Response Efficacy Beliefs,* capturing opinions that measures are effective at slowing the spread*.* Component 3 was labelled *Perceived Barriers Beliefs,* defined by the perception that protective measures are inconvenient, burdensome, and costly. Internal consistency estimates (Cronbach’s alpha) were high for Perceived Benefits (.70) and Response Efficacy (.73); however, Perceived Barriers had a low alpha of .47. This is likely due to higher variation in the three items, such that it is plausible that some people may agree with one of the items and disagree with the others.

Table A4. Items, Loadings, and Communalities for the COVID-19 Beliefs Measure

| Item | 1 | 2 | 3 | *h*2 |
| --- | --- | --- | --- | --- |
| 2. There should be a general curfew in my country (with the exception  of grocery shopping, medical treatments, and work for essential occupations) right now | **.91** | -.20 | .00 | .70 |
| 3. Risky behaviours, which might enable the spread of COVID-19, should be financially punished | **.83** | -.08 | .07 | .61 |
| 1. People in my country should cancel their participation at social gatherings right now | **.56** | .23 | -.10 | .55 |
| 9. Governments should test, track and trace every potential case of COVID-19 | **.49** | .28 | .11 | .40 |
| 8. A flatter curve means less burden on the healthcare system | -.19 | **.91** | .10 | .67 |
| 4. Social distancing is effective in slowing the spread of COVID-19 | -.02 | **.78** | -.03 | .61 |
| 7. If we don’t practice social distancing, the curve will get steeper and the number of COVID-19 cases and deaths will increase | .21 | **.66** | -.10 | .68 |
| 5. Social distancing will likely destroy our economy | .23 | .05 | **.75** | .50 |
| 10. We should rely on people getting COVID-19 in order to build up (herd) immunity | -.06 | .04 | **.71** | .52 |
| 6. Social distancing for children is unnecessary and adds burden on parents | -.13 | -.06 | **.64** | .52 |
| Component Correlations |  |  |  |  |
| 2 | .45 |  |  |  |
| 3 | -.32 | -.32 |  |  |

*Note.* Loadings above .30 are bolded; *h*^2^ = communalities

**Information Consumption Measure**

**Check News**

Participants rated the item “how often do you check the news regarding COVID-19” on a scale from 1 (never) to 5 (multiple times a day).

**News Sources**

Participants were asked which sources they use to get information about COVID-19. They rated 7 items on a scale from 1 (never) to 5 (all of the time). A principal component analysis examined the dimensionality. The scree plot and Kaiser criterion indicated two dimensions, explaining 56.86% of variance. Items and results are presented in Table A5. Component 1 was labelled *Casual Sources* and Component 2 was labelled *Official Sources.* They shared a moderate positive correlation of .05. Both subscales returned adequate reliability estimates of .66 (casual) and .74 (official).

Table A5. Items, Loadings, and Communalities for the News Sources measure

| Item | 1 | 2 | *h^2^* |
| --- | --- | --- | --- |
| 5. Word of mouth | **.82** | -.07 | .68 |
| 1. Conversations with friends/family/colleagues | **.77** | .02 | .59 |
| 7. Social media | **.72** | .03 | .52 |
| 6. News (TV, radio or internet) | **.46** | .04 | .22 |
| 3. Official international health authority websites (e.g., WHO) | .01 | **.87** | .76 |
| 2. Official Government websites | .06 | **.80** | .65 |
| 4. Scientific articles | -.05 | **.75** | .56 |

*Note.* Loadings above .30 are bolded; *h*^2^ = communalities.

**Source Check**

Participants rated one item “How often do you check that the source of information about COVID-19 is legitimate/trusted?” on a scale from 1 (never) to 5 (all of the time).

**Source Trust**

Participants rated three items about their trust in sources from 1 (strongly distrust) to 3 (strongly trust).

Items:

1. How much do you trust scientists as a source of information about COVID-19?
2. How much do you trust the media as a source of information about COVID-19?
3. How much do you trust your social circle (family friends) as a source of information about COVID-19?

Official Trust: Item 1; Casual Trust: Items 2 and 3

**Appendix B: Cross Cultural Comparison of Coping Strategies**

A series of between-subject ANOVAs were conducted to examine cross-cultural differences on the coping strategies. The results are shown in Table 9. A contrast analysis using the Tukey HSD procedure revealed numerous differences between the four countries on adaptive and maladaptive coping strategies.

Compared with UK participants, Australians reported significantly higher levels of adaptive coping strategies (humour: mean difference = 0.38, *p* < .01, 95% CI = 0.07 – 0.70; and religion: mean difference = 0.31, *p* = .03, 95% CI = 0.02 – 0.60) and lower levels of maladaptive coping strategies (denial: mean difference = -0.21, *p* = .02, 95% CI = -0.39 – -0.03; and substance use: mean difference = -0.33, *p* < .01, 95% CI = -0.58 – -0.08).

Compared with US participants, Australians reported significantly lower levels of adaptive coping strategies (planning: mean difference = -0.33, *p* = .02, 95% CI = -0.62 – -0.04; and religion: mean difference = -0.49, *p* < .001, 95% CI = -0.80 – -0.18).

Australians reported significantly lower levels of denial (maladaptive coping strategy) than participants from Canada (mean difference = 0.25, *p* < .01, 95% CI = 0.05 – 0.44).

Compared with UK participants, Americans reported significantly higher levels of adaptive coping strategies (planning: mean difference = 0.49, *p* < .001, 95% CI = 0.17 – 0.81; and religion: mean difference = 0.79, *p* < .001, 95% CI = 0.45 – 1.13) and higher levels of a maladaptive coping strategy (venting: mean difference = 0.36, *p* < .01, 95% CI = 0.08 – 0.64).

UK participants reported significantly lower levels of religion (adaptive coping strategy) than participants from Canada (mean difference = -0.57, *p* < .001, 95% CI = -0.91 – -0.23).

**Table B1. Series of ANOVAs on the Difference Between Country of Residence and Coping Strategies (df=1571).**

| Measure | Australia | Canada | UK | US | *F* | *p* | η^2^ |
| --- | --- | --- | --- | --- | --- | --- | --- |
|  | Mean (SD) | Mean (SD) | Mean (SD) | Mean (SD) |  |  |  |
| **Acceptance** | **6.63 (1.21)** | **6.39 (1.34)** | **6.56 (1.32)** | **6.42 (1.46)** | **2.93** | .03 | **.01** |
| Active | 5.01 (1.58) | 5.00 (1.5) | 4.94 (1.58) | 5.15 (1.58) | 1.03 | .38 | - |
| Behavioural Disengagement | 2.89 (1.31) | 2.95 (1.34) | 2.94 (1.28) | 3.01 (1.34) | 0.52 | .67 | - |
| **Denial** | **2.41 (0.88)** | **2.66 (1.23)** | **2.62 (1.23)** | **2.58 (1.07)** | **4.95** | **<.01** | **.01** |
| Distraction | 5.93 (1.49) | 5.89 (1.55) | 5.84 (1.53) | 5.85 (1.65) | 0.35 | .79 | - |
| **Humour** | **4.53 (1.84)** | **4.23 (1.70)** | **4.14 (1.87)** | **4.45 (1.97)** | **3.99** | **.01** | **.01** |
| **Planning** | **4.79 (1.59)** | **4.89 (1.55)** | **4.64 (1.57)** | **5.12 (1.68)** | **5.42** | **<.001** | **.01** |
| Positive Reframing | 5.16 (1.67) | 4.95 (1.69) | 4.97 (1.66) | 4.98 (1.70) | 1.61 | .19 | - |
| **Religion** | **3.10 (1.63)** | **3.37 (1.83)** | **2.79 (1.50)** | **3.59 (1.95)** | **13.54** | **<.001** | **.03** |
| Self-blame | 3.04 (1.26) | 3.12 (1.35) | 2.98 (1.29) | 3.24 (1.48) | 2.33 | .07 | - |
| **Substance Use** | **2.71 (1.34)** | **2.82 (1.42)** | **3.04 (1.63)** | **2.95 (1.55)** | **4.40** | **<.01** | **.01** |
| Use of Emotional Support | 4.43 (1.76) | 4.20 (1.74) | 4.32 (1.69) | 4.57 (1.82) | 2.53 | .06 | - |
| Use of Instrumental Support | 4.10 (1.59) | 3.95 (1.61) | 3.86 (1.56) | 4.11 (1.71) | 2.29 | .08 | - |
| **Venting** | **3.70 (1.42)** | **3.66 (1.41)** | **3.55 (1.25)** | **3.91 (1.47)** | **3.73** | **.01** | **.01** |

1. We utilised principal components analysis to maximise variance captured by the components extracted and given the exploratory nature of this stage of research. [↑](#footnote-ref-1)
